# Invisible Pursuit: A Scoping Review of Global Policy for Continuity of Care of Vulnerable Infants Under 6 Months and Their Mothers in Low- and Middle-Income Countries

**DOI:** 10.3390/children12101328

**Published:** 2025-10-02

**Authors:** Marie McGrath, Hedwig Deconinck, Stephanie V. Wrottesley, Marko Kerac, Tracey Smythe

**Affiliations:** 1 Faculty of Epidemiology and Population Health, London School of Hygiene and Tropical Medicine, Keppel Street, London WC1E 7HT, UK; marko.kerac@lshtm.ac.uk (M.K.);; 2 Emergency Nutrition Network, Kidlington, Oxford OX5 2DN, UK; 3 Institute of Health and Society, Faculty of Medicine and Health Sciences, Catholic University of Louvain, B-1200 Brussels, Belgium; 4 Department of Health and Rehabilitation Sciences, Faculty of Medicine and Health Sciences, Stellenbosch University, Cape Town 7505, South Africa

**Keywords:** infants, malnutrition, continuity, scoping, policy, vulnerable, small, newborn, underweight, paediatric

## Abstract

**Highlights:**

**What are the main findings?**

- Fragmented global policy guidance masks important synergies and gaps that hinder interdisciplinary continuity of care.
- Anthropometric markers of risk are lacking in health-oriented guidance, which weakens risk-differentiated paediatric care.

**What are the implications of the main findings?**

- There are immediate opportunities for interdisciplinary policy cooperation in global and country guidance development processes underway.
- A managed living guideline system would improve accessibility, coherence, and content of global policy for vulnerable infants under 6 months and their mothers.

**Abstract:**

Background/Objectives: Worldwide, millions of infants under 6 months are at increased risk of poor growth and development, illness, and death. We investigated the coherence of global policy characteristics, vulnerability, and continuity of care, which guides the care of vulnerable infants under 6 months and their mothers. Methods: We conducted a scoping review according to PRISMA-ScR guidance. We included English publications with no time limit, applicable to low- and middle-income countries, sourced through Google Scholar, contacts, WHO and UNICEF databases, global networks, and agency websites. The search was conducted from August 2023 to February 2024. We identified 34 documents for review. We categorised policies into guidelines, namely, WHO evidence-based recommendations and multi-source guidance documents with implementation details. We consolidated 49 vulnerability descriptors into 28 vulnerability factors and four sub-groups. We did not assess policy quality. Results: We found rich but fragmented global policy guidance. Multiple terminologies create superficial differences and mask important ones. Growth appraisal was mostly limited to nutrition-oriented guidance and was lacking in health-centric documents. Continuity of care lacked scope and depth. WHO policies are out of sync with each other and the latest evidence on mortality risk markers. WHO procedures need to accommodate non-UN documents to leverage existing guidance potential. A living policy system to manage evidence-to-policy processes and policy interactions is needed. The WHO-INTEGRATE evidence for decision frameworks could help country-led adaptations, system-sensitive global support, and WHO methodological development. Conclusions: There are immediate opportunities for interdisciplinary policy cooperation. Action is urgently needed to secure coherent evidence-based policies for equitable and effective care.

## 1. Introduction

Many infants are born vulnerable or become so in the first 6 months of life, leading to poor growth and development, increased risk of illness and death, and long-term limitations for their health, educational, and economic potential [1,2]. The burden is high in low- and middle-income countries (LMICs), where an estimated 10.3 million (17.4%) infants under 6 months (u6m) are underweight, 9.2 million (15.5%) are wasted, and 11.8 million (19.9%) are stunted [3]. Worldwide, 8.9 million infants (14.6%) are born with low birth weight (LBW) each year [4], who are at even greater and continued risk, especially those born too early or too small (premature/small for gestational age) [5].

Vulnerable infants u6m present to health and nutrition services in different ways, such as newborns with LBW who are preterm or small for gestational age; malnourished infants who are identified as wasted, stunted, underweight, or with poor growth; infants with acute or chronic illness, disability, or development concerns; and infants whose mothers have nutrition, physical or mental health, or social challenges. Providing comprehensive continuity of care to meet risk-differentiated and complex needs of mother–infant pairs involves multiple disciplines and connections within and across systems of health, nutrition, and social care.

The WHO provides evidence-based guidelines [6] and other normative products that guide member states on their public health decisions and actions [7]. Examples applicable to vulnerable infants u6m and their mothers include WHO guidelines on malnutrition [8], maternal and newborn care [9], LBW and preterm infants [10], and Integrated Management of Childhood Illness (IMCI) [11,12]. The WHO and many other United Nations (UN), non-governmental (NGOs), and civil agencies assist national authorities in implementing these recommendations in their respective contexts. Initiatives include the development of implementation guidance and direct involvement in national policy update processes. WHO is moving towards a living guideline approach, enabling responsive updates to specific recommendations as evidence emerges [13].

Understanding the nature of global policy guidance can inform both national policymakers’ appraisal of global policy developments and those supporting their efforts on how best to do so [14]. To facilitate this, we undertook this scoping review to investigate the nature and coherence of global policy that guides the care of vulnerable infants u6m and their mothers. We examined global policy content to answer four questions:

1. What are the characteristics of global policy guidance related to vulnerable infants u6m and their mothers?
2. How is vulnerability in infants u6m and their mothers described?
3. How is continuity of care for vulnerable infants u6m and their mothers conceptualised?
4. What are emerging considerations for developing policy that addresses vulnerable infants u6m and their mothers?

There is an urgent need and immediate opportunity to defragment and update existing policies and to broker interdisciplinary and interagency cooperation to do so. A living guideline system should be prioritised by the WHO to help secure equitable and impactful continuity of care for vulnerable infants u6m and their mothers.

## 2. Materials and Methods

### 2.1. Approach

We chose the scoping review method as a recognised means of exploring and describing the breadth and depth of complex and diverse literature to gain insights into the implications for policy, research, and practice [15,16,17]. We applied the JBI updated methodology for scoping reviews [18]. This scoping review was conducted in accordance with the Preferred Reporting Items for Systematic Reviews and Meta-Analyses extension for Scoping Reviews (PRISMA-ScR) [19] (Table S1). We based the scoping review protocol on a published protocol (BMJ Open) that was also registered on the Open Science Framework (OSF) platform [Reference: https://doi.org/10.17605/OSF.IO/M4JT6 (accessed on 19 August 2025)] [20].

We used a ‘learning by doing’ approach to handle complexity, by using iterative, reflective learning to refine our thinking and adapt our processes [21]. We shared the rationale and objectives and invited collaboration from WHO staff working on related policy guidance initiatives and consultations. A practitioner lens informed decisions on how to categorise, prioritise, map, and synthesise findings. We drew on our collective expertise and external reviewers to consider what is feasible and useful for practice-informed policy interpretation, implementation, and development. A Reflexivity Statement is included in the Supplementary Materials S2 and embedded throughout this paper’s narrative [22].

### 2.2. Definitions, Terminologies, and Frameworks

The definitions and terminologies used in this review, which build upon the WHO’s glossary of terms [23], are shown in Box 1. To enhance readability, we sub-categorise global policy guidance (hereafter referred to as policies) into three groups:

Guidelines: WHO-specific documents with evidence-based recommendations,

Guidance: multi-source and multi-type documents detailing implementation, and

Enabling documents: multi-source and multi-type documents that frame implementation.

We use the term ‘vulnerable’ to encompass all descriptors of risk for infants u6m and their mothers. The term “mother” is used to denote “mother or principal caregiver,” and we understand that these may not always be synonymous in practice. We apply the term “malnutrition” to encompass wasting and oedematous malnutrition. ‘Low anthropometry’ refers to single measurements. “Poor growth” refers to sequential weight/mid-upper arm circumference (MUAC)/anthropometric index measures. We apply the WHO’s continuity of care definition [23].

We used the MAMI Care Pathway Package as a starting framework to appraise the extent to which global policy guidance supports continuity of care for vulnerable mother– infant pairs [24] (Figures S3a,b). This approach was collectively developed by the MAMI Global Network [25] to support continuity of care for small and nutritionally at-risk infants u6m and their mothers (MAMI) within health systems.

Box 1 **Definitions, classifications, and terminology used in this review.**

**Policies** include normative WHO guidelines and related products and non-WHO implementation or programme guidance, briefs, training guides, manuals, action plans, strategies, and frameworks related to the care of vulnerable infants u6m and their mothers. Sources include United Nations (UN) agencies, professional networks, non-governmental organisations (NGOs), and civil society.

* *

A **guideline** is any information product developed by the WHO that contains evidence-based recommendations for clinical practice or public health policy. Recommendations are statements designed to help end-users make informed decisions on whether, when, and how to undertake specific actions, such as clinical interventions, diagnostic tests, or public health measures, with the aim of achieving the best possible individual or collective health outcomes.

* *

**Guidance** is any information product that describes how to support implementation. This includes implementation or operational guidance and manuals. It may be produced by the WHO or other entities.

* *

**Enabling documents** include frameworks, action plans, strategy papers, and training materials that support quality implementation, the development of competencies, the achievement of set goals, improvement in quality, the strengthening of political commitment, the creation of opportunities to accelerate spread, and sustainable scale-up.

* *

**Vulnerable mother–infant pairs** includes, but is not limited to, infants from birth to 6 months of age (u6m), including newborns born with low birth weight (LBW) or born premature or small for gestational age; infants with wasting or nutritional oedema (acute malnutrition), stunting, or underweight; infants with acute or chronic illness and disability or other growth and development concerns; and infants of mothers who are vulnerable because of poor physical or mental health or nutritional or social conditions.

* *

**Care**, for the purpose of this review, covers physical and mental health and nutrition care for infants u6m and physical and mental health, nutrition, and social care for their mothers, across inpatient, outpatient, and community settings.

* *

**Continuity of care** is used to indicate one or more of the following attributes of care: (1) provision of services coordinated across levels of care–primary care and referral facilities, across settings and providers; (2) provision of care throughout the life-cycle; (3) care that continues uninterrupted until the resolution of an episode of disease or risk; and (4) coherent and interconnected healthcare events over time and consistent with people’s health needs and preferences. We considered qualityrespectful care an inherent component of all attributes for person-centred care.

* *

**Person-centred care** consciously adopts the perspectives of individuals, families, and communities as participants in and beneficiaries of trusted health systems. Such perspectives include respect for persons’ values, preferences, and expressed needs regarding the coordination and integration of care; information, communication, and education; physical comfort, emotional support, and alleviation of fear and anxiety; involvement of family and friends; and transition and continuity.

### 2.3. Eligibility Criteria

We used the ‘population, concept, and context’ (PCC) mnemonic to create the eligibility criteria [19].

Population: Newborns and infants u6m who were born or became vulnerable and their mothers in LMICs.

Concept: Documents that provided global policy guidance on infant health and nutrition, child development, maternal physical and mental health, nutrition, and childcare and feeding practices, for example, support for breastfeeding and clinical and public health interventions for vulnerable infants u6m and their mothers from birth, active growth monitoring, food or supplementation interventions, and continuity of care across services and over time.

Context: We considered all settings and contexts for the care of vulnerable infants u6m and their mothers in LMICs, including but not limited to primary, secondary, and tertiary healthcare settings that may involve inpatient, outpatient and community-based services, including within the home.

We excluded regional or national policies as we sought policy guidance applicable to multiple settings and contexts (for exceptions that emerged, see Boundaries and Limitations). Due to capacity constraints, we included documents in English only. We did not set a time limit for policy inclusion.

### 2.4. Information Sources

We sourced publicly available documents through Google Scholar, personal researcher files (MM and HD), individual contacts, global health and nutrition networks, and UN/NGO websites from August 2023 to the end of February 2024. The Google Scholar search was conducted in January 2024. We sourced data from two publicly available databases, namely, the WHO Institutional Repository for Information Sharing (IRIS) [26] and the UNICEF data repository [27], along with six international agency websites and direct contacts. We hand-searched document references and snowballed to further source content. We followed up with 42 individuals identified through professional contacts and open calls to communities of practice (MAMI Global Network, CORE Group, and Global Nutrition Cluster). The professional and agency affiliations of contacted individuals are included in Tables S4a,b. The policy document database (in Excel) is available upon request. The documents we included in this review are available online on the Tableau Public platform [28].

### 2.5. Search Strategy

We explored global policy from multiple sources relating to the care of vulnerable infants u6m and their mothers. We first searched for global policy guidance related to health, nutrition, child development, and care for vulnerable infants u6m in English, without date restrictions. We considered the source, official categorisation, and nature of their content. Three researchers were involved in the process (M.M., H.D., and S.V.W.). The search strategy is included in the Supplementary Materials S5.

### 2.6. Selection of Sources

**Figure 1:**
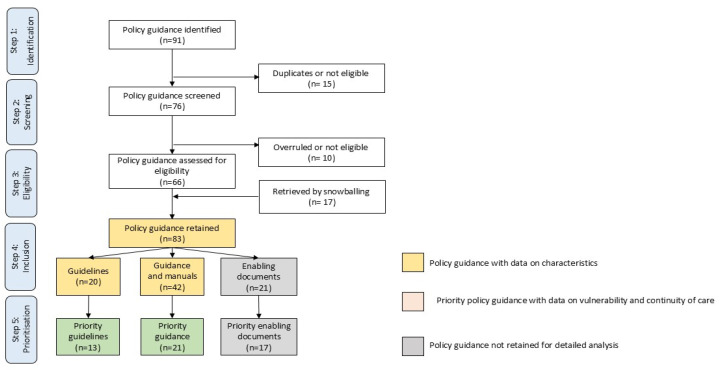
Flowchart of selection of global policy for vulnerable infants u6m and their mothers.

We examined and identified policy documents in five steps (Figure 1), as follows:

Identification: Compiled citations from grey literature searches and key contacts in an Excel spreadsheet and removed duplicates (M.M.: frame, S.V.W.: compile, and M.M. and H.D.: review and confirm).

Screening: Screened titles and summaries to exclude irrelevant policy and resolve inconsistencies and decision-making through team discussions (M.M., H.D., and S.V.W.: confer and confirm).

Eligibility: Screened full texts and removed discrepancies or outdated policy and addition of missed documents (SVW: screen; M.M. and H.D.: review and confirm).

Inclusion: Classified the retained global policy into (a) guidelines, (b) guidance, and (c) enabling documents (S.V.W. and H.D.: compile; M.M.: review and confirm).

Prioritisation: Identified policy that specifically addressed vulnerability in infants u6m and, from that, addressed healthy newborns or general, promotive infant or maternal health (M.M. and H.D.: confer and confirm).

Data screening and selection to source priority policies involved three phases within Steps 4 to 5 (Figure 1):

In Phase 1, we appraised the characteristics of 83 retained policy documents: 20 guidelines, 42 guidance documents, and 21 enabling documents. We excluded 21 enabling documents.

In Phase 2, we conducted a more in-depth exploration of the nature and coverage of vulnerability factors in 62 retained guidelines and guidance documents. We identified 34 priority policies.

In Phase 3, we appraised 34 priority policies for coverage and content regarding vulnerability factors and continuity of care.

In May 2025, we further appraised data in response to newly published evidence on mortality risk markers for infants and children [29].

### 2.7. Data Charting Process and Characteristics

Data were charted using customised forms (Microsoft Excel). Throughout the process, we iteratively organised information and adjusted our guiding frameworks accordingly.

In Phase 1, H.D. extracted key characteristics for each study, using a custom form (MS Excel). Data included the publication year, URL, source, title, type, aim, topic, target audience, target population, infant descriptors, maternal descriptors, sector, level of care, care service, age timeline, infant risks, maternal risks, interventions, and reviewer notes. M.M. independently reviewed the extracted data. Discrepancies were discussed and resolved by M.M., H.D., and S.V.W.

We extracted content on the speciality or discipline (e.g., child health, child nutrition, child development, maternal physical health, maternal mental health, maternal nutrition, reproductive health, and neonatal health) and on the sector/level (e.g., tertiary, secondary, primary, or community care).

We extracted content on interventions, e.g., active case finding or screening, health assessment, breastfeeding or non-breastfeeding assessment, breastfeeding or non-breastfeeding support, clinical care, nurturing care, Kangaroo mother care, early childhood development, crying and sleep counselling, mental health counselling, social support, follow-up visits, home visits, family involvement, and continuity of care.

We extracted content on conditions, such as health problems, diseases, disorders, injuries, disabilities, or circumstances.

### 2.8. Vulnerability

In Phase 2, for infant vulnerability, we explored content on preterm, LBW, small for gestational age, low anthropometry, poor growth, feeding problems, metabolic problems, excessive crying, disability, and acute or chronic illness. For maternal vulnerability, we looked for nutritional risk, impaired breastfeeding conditions, physical health, maternal mental health, and mothers who are multipara, primipara, adolescent, absent, or dead.

We identified 49 vulnerability factors in policy documents. Since some were very similar, we merged these into 28 vulnerability factors. To help navigate data and findings, we categorised the 28 vulnerability factors into four sub-groups: (1) poor birth outcomes (includes small and sick newborns), (2) low anthropometry or poor infant growth, (3) other infant risk factors, and (4) other maternal risk factors.

In Phase 3, we categorised and mapped 24 vulnerability factors across 4 sub-groups by policy type. We divided vulnerability factors at the median (equal to or above [≥] the 50% threshold) to determine which factors were most frequently covered. We examined the coverage of three mortality risk markers—low weight-for-age z score (WAZ), history of LBW or preterm birth, and not breastfeeding [29].

### 2.9. Continuity of Care

From our baseline MAMI Care Pathway framework, we iteratively identified 11 care dimensions, specifically, care across time, services, and levels of care; integrated care pathway; comprehensive person-centred care; early childhood development; mother, father, and family support; community participation; embeddedness; local health system support; monitoring and evaluation of services; wider multisectoral support; and organisational capacities, including resilience. A dimension fully or partially addressed was classified as ‘applied’. We mapped the relevant condition for each. Finally, we mapped which care dimensions and conditions were covered by each policy.

### 2.10. Data Items

The data items used to appraise sources in Phases 1–3 and to chart data are available in the Supplementary Materials S6. The variables comprised characteristics, vulnerability descriptors, and continuity of care dimensions. We explored the application of a life-cycle dimension but found that it oversimplified and risked misrepresentation of content (e.g., a guidance document may state a greater age range than was detailed in the guidance content). We did not appraise the quality of guidance but applied components of the AGREE II (Appraisal of Guidelines for Research and Evaluation) checklist relevant to policy development processes (scope and purpose, stakeholder involvement, and whether a procedure for update was provided) [30,31].

### 2.11. Synthesis of Results

The results were narratively synthesised, supported by summary tables and the Supplementary Materials.

### 2.12. Sharing of Results

From 2024 to the current date, data and scoping findings were shared with the WHO and UNICEF via the MAMI Global Network (co-chaired by M.M. and M.K.) in a report [32] and with policymakers in Ethiopia, who are undertaking national malnutrition policy updates (M.M.).

## 3. Results

### 3.1. Search Results

Initial searches sourced 67 potential documents: 78% (n = 52) from databases, 18% (n = 12) from agency websites, and 4% (n = 3) from Google Scholar. Most database documents were sourced from the WHO Iris Repository (85%, n = 44). Over half of website-sourced documents (n = 7, 55%) were located from six NGO websites, with one-third directly sourced from the WHO website (36%, n = 4), and 9% (n = 1) from the UNICEF website. Of website-sourced documents (n = 12), three agencies were hosts to six collectively produced policy documents—Quality of Care Network, WHO host; Healthy Mothers Healthy Babies Consortium, UNICEF host; and the MAMI Global Network, ENN host. A further 24 documents were sourced for appraisal through snowballing references, contacts, and researchers’ personal files to yield a total of 91 eligible policy documents. From these, we shortlisted and characterised sixty-two policies, which consisted of 20 guidelines [10,33,34,35,36,37,38,39,40,41,42,43,44,45,46,47,48,49,50,51] and 42 guidance documents [12,24,52,53,54,55,56,57,58,59,60,61,62,63,64,65,66,67,68,69,70,71,72,73,74,75,76,77,78,79,80,81,82,83,84,85,86,87,88,89,90,91]. From these, 34 priority policies were eligible for review, which consisted of 13 guidelines [10,33,34,35,36,37,38,39,40,41,42,43,44] and 21 guidance documents [12,24,52,53,54,55,56,57,58,59,60,61,62,63,64,65,66,67,68,69,70].

### 3.2. Characteristics

#### 3.2.1. Sources

All 13 guideline documents were published by the WHO in Geneva, except one from the regional Pan American Health Organization [37]. Twenty-one guidance documents were produced by a wide range of organisations, led/co-led by UN agencies (WHO, n = 12, 57%; UNICEF (n = 5, 24%), NGOs (n = 2, 10%), specialist agencies/foundations (n = 2, 10%), academics (n = 1, 5%), and one network involving multiple agencies/individuals (5%).

One guideline document had a direct WHO operational guidance counterpart [34,53]. While other guideline documents did not have a direct counterpart, we identified content consistent with guideline recommendations both within and beyond UN-authored guidance, e.g., for early childhood development [38,60], LBW/preterm [10,55,63], and malnutrition/poor growth [24,33]. In some cases, such guidance preceded the WHO recommendations for which it provides relatable implementation details [24,33].

#### 3.2.2. Publication Dates and Updates

Publication dates ranged from 2012 to 2023 for guideline documents and from 2003 to 2022 for guidance documents. Two guideline documents (15%) specified an update date, and six made no reference to an update (19%). Over one-third (n = 5, 38%) recorded a less specific intent, such as updates with emerging evidence, in line with other guidance developments, or stated that they may consider it in the future. Six guideline documents (46%) included a named or departmental contact. Amongst the guidance documents, only two documents (10%) specified an update date (year), one indicated the intent, and eleven (52%) provided a contact person or department.

#### 3.2.3. Conditions

Guideline documents covered four conditions related to newborn care, six on illness, one on low anthropometry and poor growth, one on breastfeeding, and one on early childhood development. Guidance documents covered five conditions on newborn care, seven on illness, two on preterm and LBW, two on poor growth and malnutrition, one on breastfeeding, one on feeding difficulties, one on perinatal mental health, and one on reproductive health.

Some guideline documents targeted a single condition (e.g., tuberculosis) [34,35,36,40,41,43] or a set of conditions (e.g., common childhood illness and poor growth and development) [33,44]. Others targeted specific vulnerability profiles (e.g., LBW newborns) [10,37,42] or embedded content specific to them within a broader condition/population policy [38,39]. The care delivery point ranged from inpatient facilities (e.g., resuscitation) to the community (e.g., outpatient facilities) and home levels (e.g., breastfeeding support).

Guidance documents targeted single conditions (e.g., cerebral palsy) [53,68] or sets of conditions (e.g., birth impairments) [12,64,66], interventions (e.g., Kangaroo mother care and early childhood development) [59,60,61,62], and profiles of vulnerability (e.g., small sick infants and preterm infants) [63,65,69]. Some targeted multiple at-risk infants u6m [24,60] or subsets of these, e.g., preterm/LBW newborns [55]. Some guidance documents focused on quality of care and behaviours or factors that influence vulnerability, e.g., feeding difficulties and maternal mental health [54,57,58,59]. One guidance document was related to service integration [56].

#### 3.2.4. Target Audience

All policy guidance included a target user. Across guideline documents, the target user was broad, diverse, and multiple across disciplines or sub-specialities within health and nutrition, system levels, and professional remit, most often policymakers, programmers, health workers, trainers, and institutions, typically the government, UN agencies, NGOs, academics, and funders. Guidance documents were more oriented toward health practitioners, clinicians, or service planners; one-third (n = 5, 36%) specified policymakers as a target.

#### 3.2.5. Target Population

All 13 guideline documents targeted infants u6m, and 8 (61%) incorporated aspects related to mothers [10,33,35,36,37,38,39,42]. Twelve guideline documents (57%) were applicable from birth, of which three were specific to the early postnatal period [41,42,43]. One guideline document from birth limited one recommendation (nutritional supplementation) to infants from 1 month of age [33].

All 21 guidance documents covered infants u6m or a subset of them; half (n = 11, 52%) included mothers, of which one targeted perinatal mental health [54]. Nine (21%) guidance documents were related to other aspects of service provision, e.g., integration of services, monitoring and evaluation, resources and equipment, and quality of care.

#### 3.2.6. Vulnerability Descriptor for Infants

Across all policies, many descriptors were identified for infant vulnerability according to the topic, condition, and speciality, including healthy newborn, healthy infant, vulnerable newborn, born preterm, LBW, small, small sick, (non-)breastfed, developed wasting or acute malnutrition, underweight, stunting, growth faltering, at risk of poor growth and development, feeding problem, excessive crying, physical disability or developmental delay, and acute or chronic illness.

#### 3.2.7. Vulnerability Descriptor for Mothers

As with infants, many descriptors were identified for maternal vulnerability, including healthy, sick, mental health issue, malnourished, adolescent, prenatal, perinatal, postnatal issues, (not) lactating, multipara, primipara, absent, and dead. Female-headed households were not identified as a vulnerability in any policies.

#### 3.2.8. Sector

Guideline development was led by specialists in child health (n = 9, 69%), involving sub-specialities (neonatal care (n = 6, 46%), urgent care (n = 3, 23%), disease specialists (n = 3, 23%), and maternal health (n = 2, 15%)) and specialists in nutrition (breastfeeding and malnutrition) (n = 2, 15%) and early childhood development (n = 1, 8%). Guidance development was led by specialists in newborn care (n = 6, 29%), nutrition/feeding (n = 4, 19%), urgent care (n = 1, 5%), illness management (n = 6, 29%), disability (n = 2, 10%), maternal mental health (n = 1, 5%), reproductive health (n = 1, 5%), and inter-sectors/disciplines (n = 4, 19%).

Some guideline documents cross-referenced others; for example, the 2023 malnutrition guidelines deferred to neonatal guidance for nutritional supplementation in infants under 1 month of age [45]. Others embedded details of recommendations within their document; for example, the tuberculosis guideline document specified anthropometric criteria for severe acute malnutrition [34], and the 2023 malnutrition guidelines [33] embedded detailed elements of IMCI [12]. The WHO guideline documents did not cross-reference non-UN-produced documents.

#### 3.2.9. Level of Care

About one-third of guideline documents (n = 5, 38%) encompassed all levels of care, one-third specified facility-based care (n = 5, 38%), and one-quarter focused on primary/community care (n = 3, 23%). More than half (n = 11, 52%) of the guidance documents covered care at primary and secondary levels, 19% (n = 4) mainly covered inpatient or specialist care, and three were more generalisable across all care levels (14%).

#### 3.2.10. Care Service

Guideline documents covered neonatal health, child health, maternal health, infant and young child feeding (breastfeeding), and malnutrition treatment services to widely varying extents. This was influenced by the number and nature of recommendations and the extent of elaboration in good practice statements and notes.

Guidance documents had a similar service scope and variation as guideline documents. However, they included more specific and detailed sub-speciality or sub-group content, e.g., for early childhood development, children with disability, feeding difficulties [55,58,60,68], and more cross-sectoral comprehensive coverage of care [24,63,64].

### 3.3. Vulnerability Factors

The coverage of 28 consolidated vulnerability factors across the 34 reviewed policies is shown in Table 1 and mapped by policy document in Table S7. Sixteen factors were specific to the infant, and twelve factors were specific to the mother.

In guideline documents, factors mentioned most frequently (≥50%) were associated with poor birth outcomes (congenital illness, LBW, and preterm), breastfeeding difficulties, and illness in infants (Table 2). Factors less frequently mentioned (40–50%) were related to mothers’ physical health. In guidance, factors most frequently mentioned (≥50%) were associated with small and sick newborns (poor growth, breastfeeding difficulties, no breastfeeding, and infant illness) and the mother’s wellbeing (physical health, breastfeeding conditions, mental health, and social or contextual factors).

#### 3.3.1. Anthropometry and Growth

Around one-third of guideline documents included low anthropometry or poor growth as a vulnerability factor (n = 4, 31%) [33,34,37,44]. Of these, three included low anthropometry—one specified low WAZ [33], and two specified low weight-for-length z score (WLZ) [34,44].

Coverage was higher in guidance documents; over half included low anthropometry or poor growth (n = 13, 62%). Of these, seven (33%) included low anthropometry—two included low WAZ for infants aged 0–6 months [24,60], two IMCI guidance documents applied WAZ to infants aged 0–2 months and WLZ to infants aged 2–6m [12,64], three WHO guidance documents applied WLZ criteria [52,53,67], one applied MUAC from 6 weeks to 6 months [33], and one applied MUAC from birth [24]. Anthropometric or growth vulnerability was not included in guidance documents on disability [66,68] follow-up of at-risk newborns, including community care [63], feeding support [55], and peri-natal mental health [54].

#### 3.3.2. Feeding

Breastfeeding difficulties (related to the infant) or breastfeeding conditions (related to the mother) were commonly included in policies to identify infant vulnerability (see Table 1). Not being breastfed was included more often in guidance documents (n = 13, 65%) than in guidelines (n = 4, 31%).

#### 3.3.3. Maternal

Maternal vulnerability (e.g., breastfeeding conditions, mental and physical health, and social or contextual factors affecting care and feeding practices) was referred to less often in guideline documents (n = 10, 77%) compared to guidance documents (n = 17, 85%), as shown in Table 1. Coverage in guideline documents was highest in those related to early postnatal care. None of the guideline documents and two guidance documents [24,60] included the mother’s MUAC, while other anthropometric indicators (e.g., weight) were absent from all. Adolescent motherhood was referred to in two guideline documents (15%) and one-third of guidance documents (n = 7, 33%). Other, less commonly identified, vulnerabilities included birth complications and short birth spacing.

#### 3.3.4. Illness

Guideline documents covered four conditions related to newborn care, six on illness, one on low anthropometry and poor growth, one on breastfeeding, and one on early childhood development. Guidance documents covered five conditions on newborn care, seven on illness, two on preterm and LBW, two on poor growth and malnutrition, one on breastfeeding, one on feeding difficulties, one on perinatal mental health, and one on reproductive health.

With the exception of one guideline document on common childhood conditions [44], LBW/prematurity was not included as a vulnerability factor in clinical (sick infant care) guideline documents. All clinical guidance documents included LBW/prematurity as a vulnerability, except for tuberculosis operational guidance [53]. Overall, there was little policy content specific to infants who were born small for gestational age.

#### 3.3.5. Combination of Risk Markers

Individual and combined coverage of three risk markers for infant mortality (low WAZ, non-breastfed, and LBW/premature) was low across policies [29], as shown in Table 1. Only one guideline document [33] and two guidance documents [60] included all three risk markers for infants up to six months of age. LBW/prematurity was not specified as a risk factor in IMCI guidance, and low WAZ was recommended for infants who were 0 to 2 months only [12,64]. Clinically oriented guidance did not include WAZ; one WHO guidance included WAZ reference tables but did not specify weight assessment and appraisal as part of case management [67].

### 3.4. Continuity of Care

Table 3 maps each policy document across 11 dimensions of continuity of care and by condition. The coverage of dimensions varied greatly across guidelines and guidance. Table 4 summarises the coverage of continuity of care by policy type and provides a summary appraisal of findings for each dimension. There was high coverage across several policy documents, some that were condition-specific, such as on perinatal mental health [54], and some that were population-specific, such as hospital care for small, sick and preterm newborns [59]. However, we found that high coverage often reflected intent but did not equate with in-depth content to provide guidance on how to achieve this. Implementation details were more often related to management continuity for conditions within services, with limited coverage of operational and wider multisectoral aspects of continuity of care.

## 4. Discussion

In this review, we examined global policy content related to the care of vulnerable infants u6m and their mothers. In our discussion, we explore the implications of our findings regarding coherence, opportunities, and gaps within and across policies. We consider policy content in relation to policy context, actors, and guidance development processes [92]. We identify strategic and practical directions for enhancing policy-directed care guided by national policymaker interests [18].

### 4.1. Policy Content

#### 4.1.1. Characteristics

Our review revealed a global policy landscape that is rich and diverse but also highly fragmented. Policies span multiple disciplines and development processes, resulting in variable content both across and within guidance documents over time. We identified a wide range of vulnerability descriptors that, while seemingly distinct, often refer to overlapping populations. This creates artificial distinctions and risks obscuring opportunities for alignment. For example, a “small vulnerable newborn” who may be later identified as “malnourished” or “underweight” is often treated as a different case in policy, despite being the same child clinically. Conversely, terminological similarities can obscure important differences. The 2023 WHO malnutrition guideline, for instance, expanded its scope from severely malnourished infants u6m [8] to infants u6m at risk of poor growth and development [33]. However, while the population coverage has broadened, some 2013 recommendations—such as those on antibiotic use—were not updated. The existing 2013 recommendation for antibiotics still stands, although it is not transferable to the wider group of at-risk infants that are now included in the guideline. These inconsistencies undermine policy coherence, reduce content predictability, and hinder accessibility. They create a layer of complexity that risks confusion for implementers and mask both critical policy gaps and inter-policy potential.

#### 4.1.2. Vulnerability of Infants

Poor infant growth and low anthropometry were underrepresented as markers of vulnerability in the policies reviewed. Growth and anthropometric indicators were more frequently addressed in nutrition-oriented policy guidance. In most postnatal guidance, LBW is treated primarily as a risk factor for an acute event (birth) rather than the starting point for continued growth monitoring and appraisal. This likely reflects the narrow life-course focus of many postnatal policies, which tend to prioritise the high-risk neonatal period.

Clinically oriented guidance sometimes addresses breastfeeding difficulties but includes growth assessment less commonly. Underweight is recommended as a risk criterion in only one WHO guideline [33], is limited to infants under two months in IMCI guidance [12,64], and is absent from WHO clinical policy documents. These omissions are not aligned with the current evidence, which shows that underweight infants u6m—particularly those with comorbidities requiring inpatient care—face substantially higher mortality risk [29]. This discrepancy may stem from a prevailing perception that anthropometric indicators are specific to nutrition, rather than being recognised as sensitive predictors of mortality. Addressing this gap may require a conceptual shift—one that reframes anthropometric measures as key indicators of vulnerability across health domains, not just within nutrition. This is necessary to ensure management embeds and extends beyond nutrition interventions.

#### 4.1.3. Vulnerability of Mothers

Maternal health was commonly considered in policy guidance. However, the content was inconsistent and typically framed in relation to infant needs, rather than from the mother’s perspective. There was limited coverage of maternal nutritional status, anaemia, and anthropometry. Postnatal guidelines offered greater detail on maternal components, which may reflect how mothers’ wellbeing is inherent in early antenatal care. However, similar to others, we found that this tapered off thereafter [93]. Where maternal care was embedded as a core component of at-risk infant care [24,33,60], implementation details remained weighted towards infant needs. The limited coverage of maternal needs beyond the immediate postnatal period may reflect the typical segregation between adult and child health services. These policy gaps may also reflect broader, systemic shortcomings in providing adequate provision for women’s health and nutrition [94,95]. If so, even if policies improve, implementation requires services that may be inconsistent or non-existent.

These limitations have far-reaching implications, not only for infant health but also for the rights and wellbeing of women themselves. It is well established that a mother’s physical and mental wellbeing influences her ability to nourish and care for her infant, as well as to maintain her own health. However, care for an at-risk infant draws heavily on a mother’s mental and physical resources. She is a woman who has her own vulnerabilities and needs. Safeguarding and strengthening a mother’s nutrition and health status is essential for delivering responsible, equitable, and effective care [96,97].

#### 4.1.4. Continuity of Care

Continuity of care was frequently cited as a guiding principle in the policies reviewed, but it was often not well operationalised. Policy content tended to focus on the management of specific conditions—such as feeding difficulties or acute illness—with much lower coverage of the informational and relational components of person-centred care [98]. This limitation may partly reflect the scope of policy guidance itself. For example, WHO guidelines provide evidence-based recommendations for selected aspects of care for a particular population or condition, which may not extend to broader, cross-cutting components of continuity of care.

Policy guidance for vulnerable newborns was primarily concentrated on the early postnatal period, with a strong focus on breastfeeding and clinical care. However, there was limited guidance on follow-up into later infancy and early childhood. Infants born small for gestational age received minimal policy attention. While some nutrition and health policies mention follow-up for babies born too small or too early, overall policy coverage remains limited. Notably, low birth weight and prematurity are not recognised as risk markers within IMCI guidance [12,64].

We also observed areas of strong synergy across policy documents. In some cases, these links were explicit; for example, the WHO’s tuberculosis guidelines are directly supported by derivative operational guidance. In others, cross-references were embedded within policy texts—for instance, the 2023 updated WHO malnutrition guidelines cross-referenced neonatal care guidance for very young infants. However, some synergies remained implicit. The MAMI Care Pathway Package, for example, aligns closely with the 2023 malnutrition guideline recommendations but is not directly referenced within them.

These findings highlight missed opportunities for inter-policy alignment to support more coherent and continuous care for vulnerable infants and their mothers. Small vulnerable newborns become small vulnerable babies, but this is not recognisable in policy content [5,99]. Realising this potential will require more intentional integration and bridging of related guidance across sectors and life-course stages.

### 4.2. Policy Processes

Our findings illustrate that policy coherence requires policy connectivity. We found, like others have, that there are practical challenges in applying the WHO’s living guideline approach to more complex fields of care [100]. For example, updates to even one recommendation require cascading revisions across multiple UN and non-UN documents. The living guideline approach provides a means for responsive updates, but so far, it has only been applied to some policy guidance, such as COVID-19 [101,102], and there is no official guidance on how to implement it [13]. We identified immediate needs in this regard. For example, a recent secondary data analysis identified three “readily assessable” indicators (non-breastfed, LBW/premature, underweight) to inform risk-differentiated care in services [29]; yet, all three had low coverage across WHO and non-UN guidance. There are also outstanding needs; for example, WHO clinical guidelines only reference WHZ < −3 from the 2013 malnutrition guideline, which is now outdated [29]. Furthermore, no mechanism exists to systematically identify where inconsistencies and gaps may lie in what already exists—recognising and identifying the extent of a problem is essential to figuring out a solution.

### 4.3. Ways Forward

Our findings reflect the inherent complexity of real-world policy and implementation contexts and interactions [103]. Improving coherence requires approaches that recognise and embrace this rather than attempt to mute it. The challenge lies in how to interplay policy, evidence, and practice without furthering complications or heightening resource demands. Working with what exists and considering what is needed, we make several suggestions to strengthen policy coherence immediately and in the future.

#### 4.3.1. Toward a Living Policy System

Our findings suggest the need for a dynamic, living policy system to improve accessibility, identify synergies across disciplines, and support the alignment of policy processes. We envision an interlinked and continuously updated policy curation mechanism, framed around person-centred continuity of care for vulnerable infants u6m and their mothers. This umbrella framework could help situate diverse policy guidance within the broader context of care continuity, both within and across sectors. Active policy surveillance would enable timely updates in response to emerging evidence and facilitate the dissemination of policy addenda or revisions. Integrated feedback mechanisms could help identify and respond to the priorities and needs of national policymakers, ensuring that global guidance remains relevant and valued. Embedding learning processes within this system could also support the implementation of ‘living guidelines’ for complex care scenarios [13,100] while contributing to an improved evaluation of the impact of normative guidance [104].

Two existing WHO initiatives offer promising models for such an approach. First, the WHO Clinical Care in Crises (CCC) mobile app, developed for frontline workers [105], used an international advisory group to identify and address policy inconsistencies to produce algorithms. Second, the COVID-19 Living Guidelines mechanism—a collaboration between the WHO, the MAGIC Evidence Ecosystem Foundation, and *The BMJ*—uses a standing expert panel to produce real-time living network meta-analyses, with regular updates and alerts [102] hosted on the MAGICApp platform [106]. Several UN guidelines we reviewed are already hosted on MAGICApp. However, what remains lacking is a comprehensive, curated collection of interconnected policy content, structured under a unified framework and actively managed as a living system.

#### 4.3.2. Handling Complexity Within Policy Guidance

Our review suggests that methodological rigour alone does not ensure consistency in guideline outputs [6]. The significant variation we observed across WHO guideline content likely reflects attempts to address the “whether, when, and how” of delivering complex care within the constraints of condition- or population-specific guidance. Emerging frameworks, such as WHO-INTEGRATE [107], offer valuable tools for managing complexity of the GRADE (Grading of Recommendations Assessment, Development and Evaluation) evidence to decision-making processes [6]. The variability we observed across guidance also reflects the varied sources and approaches of many stakeholders in developing content. Broader applications of evidence to quality assurance frameworks, such as AGREE and WHO-INTEGRATE, enhance the rigour of both UN and non-UN content, support greater policy coherence, and contribute to methodological development and its evaluation [108,109].

Implementation guidance developed at the global level will inevitably have limitations when it comes to contextualising it to diverse and dynamic settings. National stakeholders are best positioned to adapt global guidance by tailoring recommendations to their local population needs and system capacities. Incorporating frameworks like WHO-INTEGRATE into national adaptation processes could help support national decision-making by helping to navigate uncertainty and balance trade-offs. WHO regional and country offices could facilitate this, for example, by deploying roving system specialists to foster cross-country learning and collaboration. Furthermore, the WHO could benefit from systematically learning from country-led adaptations. Insights from these experiences could inform the organisation’s own evidence-to-policy methodologies and contribute to institutional impact evaluations of normative guidance development [110].

#### 4.3.3. Investing in Policy Cooperation

Ongoing policy processes at the WHO present a timely opportunity to strengthen interdepartmental collaboration, align outputs, and support national policy development. For example, current guidance development work—spanning malnutrition, care for small and sick newborns, and IMCI—offers a concrete opportunity to promote policy coherence and ensure consistent, aligned content. While inter-policy collaboration within the WHO often occurs informally, such ad hoc coordination requires considerable time and effort. To enhance the efficiency and impact, an institutionalised mechanism that mandates and resources interdepartmental coordination as a core function is needed. Given increasingly constrained national and global resources, streamlining processes across disciplines and departments offers clear strategic and economic advantages [111,112]. Leveraging existing tools and evidence represents a strong investment case.

Importantly, the WHO is not the sole source of public health guidance. Content produced by non-UN actors can also meet rigorous standards and be highly relevant. Our review identified several opportunities for cross-agency policy cooperation. For instance, the MAMI Care Pathway Package applied AGREE criteria and was developed through expert-peer collaboration. It aligns with the WHO’s 2023 malnutrition guidelines, is modelled on and extends the IMCI guidance, and incorporates outpatient follow-up for vulnerable newborns. The WHO has recognised it as a “concrete illustration” of how to operationalise recommendations for risk-differentiated care [113], and results from an evaluated randomised controlled trial (RCT) in Ethiopia are expected imminently. However, WHO procedures do not currently provide a clear mechanism for incorporating existing non-UN guidance that does not follow WHO-type formal procedures [6,114]. The WHO’s ‘guidance on guidelines’ does not specify the appraisal of existing guidance and tends to assume that implementation guidance is derived from WHO recommendations. Our review found that, in some cases, non-UN guidance supporting WHO recommendations preceded the guideline’s publication. Updating the WHO’s procedures to also consider high-quality non-UN guidance could help address urgent policy gaps, ease the burden on the WHO’s own guideline development processes, and reduce duplication of effort.

#### 4.3.4. Shared Problems May Spark Collective Solutions

Multiple descriptors of vulnerability risk creating artificial distinctions between what are often interconnected challenges. Siloed framing contributes to siloed policies that risk siloed services. Inconsistent definitions and fragmented guidelines have hindered action on LBW within the reproductive health field [115]; our review suggests that similar consequences are likely in the broader mother–infant health landscape. Process-generated differences may fuel sectoral competition, national confusion, and missed opportunities for integrated care. While agreeing to common terms across stakeholders is likely unrealistic, securing shared understanding and pursuing common goals is. A clearly defined shared problem could help consolidate efforts, maximise existing tools, and attract attention and resources that are typically insufficient across health and nutrition domains [116].

#### 4.3.5. Strengths and Limitations

This scoping review focused on global policy guidance and did not assess evidence from trials or interventions. We did not evaluate the risk of bias or the quality of policy documents, as these are beyond the remit of scoping reviews. We excluded enabling policy guidance that lacked sufficient depth for our practice-centred focus. Ethical approval was not required, as we used only publicly available data.

We reviewed only English-language publications. Four regional/national documents were included due to their global relevance [37,60,65,67], but as we did not systematically source these, others may have been missed. We used our professional networks to source policies, which may have introduced selection bias. We did not formally appraise policy context, stakeholders, and processes. Stakeholder consultation would have strengthened the review, but it was not feasible; this remains an important area for future research (21). Future reviews should also seek documents in other languages and investigate the extent of translation of global guidance produced in English. Our own experience, assumptions, and relationships shaped this review, potentially introducing bias, but also adding value.

## 5. Conclusions

We found a rich melting pot of valuable policy guidance relevant to the care of vulnerable infants u6m across sectors and specialities. Global policy guidance spans multiple disciplines, requiring improved transparency, coherence, and collaboration to manage its inherent complexity. There is both the need and opportunity to defragment existing policies, develop inter-specialist understanding, and secure inter-sectoral policy pathways of care. There are inter-policy synergies to leverage and urgent gaps to address regarding growth, follow-through care for small vulnerable newborns, and maternal nutrition and health. The COVID-19 living guideline mechanism exemplifies the value of policy navigation. It is time to match the same ambition and investment to vulnerable infants and their mothers. National leadership to contextualise global policy content will maximise the potential for positive policy impact. These actions are an economic and equitable imperative for securing the best possible care for vulnerable infants u6m and their mothers.

## Figures and Tables

**Table 1:** Vulnerability factors mapped across the 34 policy documents by policy guidance type.

Vulnerablilty Factors	Guideline Documents *		Guidance Documents		All Policy Documents	
n = 28	n = 13	(%)	n = 21	(%)	n = 34	(%)
*Poor birth outcomes*						
Congenital illness	7	(54)	15	(71)	22	(65)
Low birth weight (LBW) **	7	(54)	14	(67)	21	(62)
Preterm **	7	(54)	13	(62)	20	(59)
Disability or congenital abnormality	5	(38)	12	(57)	17	(50)
Birth trauma or complications	5	(38)	7	(33)	12	(35)
Preterm morbidity	4	(31)	11	(52)	15	(44)
Small for gestational age	3	(23)	5	(24)	8	(24)
*Low anthropometry or poor growth*						
Low anthropometry (low WAZ **, low WLZ, or low MUAC)	3	(23)	7	(33)	9	(26)
Poor growth (weight loss or stagnant or insufficient weight gain)	3	(23)	12	(57)	15	(44)
Nutritional oedema	3	(23)	6	(29)	9	(26)
*Risk factors related to the infant*						
Breastfeeding difficulties	7	(54)	14	(67)	21	(62)
Illness	7	(54)	11	(52)	18	(53)
Not breastfed **	4	(31)	13	(62)	17	(50)
Neurodevelopment concerns	4	(31)	11	(52)	15	(44)
Hospitalisation history	1	(8)	5	(24)	6	(18)
Mental health	0	(0)	3	(14)	3	(9)
*Risk factors related to the mother*						
Physical health	6	(46)	15	(71)	21	(62)
Breastfeeding conditions	5	(38)	12	(57)	17	(50)
Mental health	5	(38)	11	(52)	16	(47)
Social or contextual factors affecting care and feeding practices	3	(23)	11	(52)	14	(41)
Birth complication	3	(23)	6	(29)	9	(26)
Adolescent	2	(15)	7	(33)	9	(26)
Absent or died	2	(15)	5	(24)	7	(21)
Multipara	1	(8)	6	(29)	7	(21)
Primipara	1	(8)	2	(10)	3	(9)
Anaemia	0	(0)	3	(14)	3	(9)
Birth spacing	0	(0)	2	(10)	2	(6)
Low MUAC	0	(0)	2	(10)	2	(6)

* In descending order by frequency in guideline documents. ** Risk markers for increased infant u6m mortality identified in the published analysis [29].

**Table 2:** Vulnerability factors present in at least half of the 34 policy documents.

Category of Vulnerability	Guideline Documents (>50% Coverage)	Guidance Documents (>50% Coverage)
Poor birth outcomes	Congenital illness Low birth weight Preterm	Congenital illness Low birth weight (LBW) Preterm Disability and/or congenital abnormality Preterm morbidity
Low anthropometry or poor growth	No	Poor ponderal growth
Risk factors related to the infant	Breastfeeding difficulties Illness	Breastfeeding difficulties Not breastfed Illness Neurodevelopment concerns
Risk factors related to the mother	No	Physical health Breastfeeding conditions Mental health Social or contextual factors

**Table 3:** Coverage of continuity of care dimensions and related conditions across 34 policy documents.

Title of Document	Condition	Care Across Time, Services, and Levels of Care	Integrated Care Pathway	Comprehensive Person-Centred Care	Early Childhood Development	Mother, Father, and Family Support	Community Participation	Embeddedness (Mainstream)	Local Health System Support	M&E	Wider Multisectoral Support	Organisational Capacities
**Guidelines (blue)**												
2023 WHO Guideline on the prevention and management of wasting and nutritional oedema in infants and children under 5 years	Poor growth and development	✔️	✔️	✔️	✔️	✔️	-	✔️	-	-	✔️	-
2022 WHO Consolidated guidelines on tuberculosis in children and adolescents	Tuberculosis	✔️	✔️	✔️	-	✔️	✔️	✔️	✔️	✔️	-	-
2022 WHO Recommendations for care of the preterm or low-birth-weight infant	Preterm and LBW	-	✔️	-	-	✔️	✔️	✔️	✔️	✔️	✔️	✔️
2021 WHO-PAHO Evidence-based clinical practice guidelines for the follow-up of at-risk neonates	Newborn care	✔️	✔️	✔️	✔️	✔️	-	-	-	✔️	-	-
2021 WHO Guideline: Infant feeding in areas of Zika virus transmission	Zika virus	-	-	-	-	✔️	-	✔️	-	-	-	-
2021 WHO Consolidated guidelines on HIV prevention, testing, treatment, service delivery and monitoring	HIV	✔️	✔️	✔️	-	✔️	✔️	✔️	✔️	✔️	✔️	✔️
2020 WHO Improving early childhood development	ECD	✔️	n/a	n/a	✔️	✔️	-	-	✔️	✔️	-	-
2018 WHO Guideline: Counselling of women to improve breastfeeding practices	Breastfeeding	✔️	✔️	✔️	-	✔️	-	✔️	✔️	✔️	-	✔️
2016 WHO Paediatric emergency triage, assessment and treatment (ETAT)	Critically ill child	-	-	-	-	-	-	-	✔️	✔️	-	-
2015 WHO Recommendations on interventions to improve preterm birth outcomes	Preterm	-	-	-	-	✔️	-	✔️	✔️	✔️	-	-
2015 WHO Guideline: Managing possible serious bacterial infection in young infants when referral is not feasible	Ill child	✔️	-	-	-	-	-	✔️	✔️	✔️	-	-
2012 WHO Recommendations for management of common childhood conditions	Ill child	-	-	-	-	-	-	✔️	✔️	-	-	-
2012 WHO Guidelines on basic newborn resuscitation	Newborn resuscitation	-	-	-	-	-	-	✔️	-	✔️	-	-
**Guidance (green) and manuals (yellow)**												
2022 WHO Guide for integration of perinatal mental health in maternal and child health services	Perinatal mental health	✔️	✔️	✔️	✔️	✔️	✔️	✔️	✔️	✔️	✔️	✔️
2022 UNICEF, University of Pretoria Feeding preterm and low-birth-weight newborns	Preterm and LBW	✔️	✔️	✔️	✔️	✔️	✔️	✔️	-	-	-	-
2022 WHO-Europe Pocketbook of primary healthcare for children and adolescents	Ill child	-	✔️	✔️	✔️	✔️	✔️	✔️	✔️	-	✔️	✔️
2022 SPOON Identifying feeding difficulties in infants—guidelines for healthcare professionals	Feeding difficulties	✔️	-	-	-	-	-	✔️	-	-	-	-
2022 SPOON Screening children for feeding difficulties—guidelines for healthcare professionals	Feeding difficulties	✔️	-	✔️	✔️	✔️	✔️	✔️	✔️	-	-	-
2022 UNICEF Integrating early detection and treatment of child wasting into routine primary healthcare services	Wasting	✔️	n/a	n/a	-	-	✔️	✔️	✔️	✔️	✔️	✔️
2022 WHO Operational handbook on tuberculosis. Module 5: Management of tuberculosis in children and adolescents	Tuberculosis	n/a	✔️	✔️	-	✔️	✔️	✔️	✔️	✔️	✔️	-
2021 ENN, LSHTM, MAMI Care Pathway Package, Version 3 (guiding framework)	Small and nutritionally at-risk infant and mother	✔️	✔️	✔️	✔️	✔️	✔️	✔️	✔️	✔️	✔️	✔️
2020 WHO, UNICEF The Baby-Friendly Hospital Initiative for small, sick and preterm newborns	Breastfeeding	-	✔️	✔️	✔️	✔️	✔️	✔️	✔️	✔️	✔️	-
2020 Partners in Health, UNICEF Early Childhood Development Support for High-Risk Infants	Preterm and LBW	✔️	✔️	✔️	✔️	✔️	-	✔️	-	-	✔️	-
2019 WHO Integrated Management of Childhood Illness of the sick young infant aged up to 2 months. Chart booklet	Ill child	✔️	✔️	✔️	-	✔️	-	✔️	-	✔️	-	-
2018 IASC Field Manual on Reproductive Health in Humanitarian Settings	Reproductive health	✔️	✔️	✔️	-	✔️	✔️	✔️	✔️	✔️	✔️	✔️
2016 WHO Oxygen therapy for children	Ill child	n/a	✔️	-	-	✔️	-	✔️	✔️	✔️	-	-
2015 WHO, UNICEF, USAID Caring for newborns and children in the community: planning handbook for programme managers and planners	Newborn care	n/a	✔️	✔️	✔️	✔️	✔️	✔️	✔️	✔️	-	-
2014 WHO-Europe Hospital care for mothers and newborn babies: quality assessment and improvement tool	Newborn care	✔️	✔️	✔️	-	✔️	✔️	✔️	✔️	✔️	-	✔️
2014 WHO Integrated Management of Childhood Illness—Chart booklet	Ill child	n/a	✔️	✔️	-	✔️	-	✔️	-	-	-	-
2014 Christian Blind Mission (CBM) Recognising impairments at birth	Newborn care	n/a	✔️	✔️	✔️	-	-	✔️	✔️	-	-	-
2013 WHO Pocketbook of hospital care for children	Ill child	n/a	✔️	✔️	✔️	✔️	✔️	✔️	-	✔️	-	-
2012 CBM Cerebral Palsy	Cerebral palsy	n/a	✔️	✔️	✔️	✔️	✔️	✔️	✔️	-	-	-
2003 WHO Kangaroo mother care: a practical guide	Newborn care	n/a	✔️	✔️	✔️	✔️	✔️	✔️	✔️	✔️	✔️	-
2003 WHO Managing newborn problems	Newborn care	n/a	✔️		-	✔️	✔️	✔️	-	✔️	-	-

✔️ = care dimension covered; LBW = low birth weight; ECD = early childhood development, and n/a = not applicable.

**Table 4:** Coverage and appraisal of continuity of care across 34 policy documents by policy type.

Dimensions of Care	Guideline Documents (n = 13)	Guidance Documents (n = 21)	Summary Appraisal *
Care across time, services, and levels of care	7	10	Gaps in continuity across time, primarily for follow-up after discharge from a particular service or when infants ‘age out’ of the service without adequate referral mechanisms; gaps in continuity between services and levels of care, e.g., linking facility- and community-based care.
Integrated care pathway	6	18	Strong focus on addressing one or more condition(s), without integration into a broader care pathway that assesses and responds to maternal and infant risks and monitors progress/links to follow-up care, particularly at other levels of the health system (primary care/outpatient/community levels).
Comprehensive person-centred care	5	17	Guidelines centred on the condition rather than the person (mother or infant) and a lack of comprehensive assessment and care for both mother and infant together.
Early childhood development	3	12	Limited contextualisation within a framework that supports early child development, with a focus on the identification and management of one or more condition(s) rather than on a holistic approach to ensuring optimal growth and development.
Mother, father, and family support	9	18	Primary focus on supporting the mother in feeding and care practices for the infant, including targeted counselling for mothers and other caregivers/family members in some cases. Lack of inclusion of broader aspects of support, particularly for the mother’s own health and social circumstances.
Community participation	3	15	Limited aspects of community participation in care/organisation of care, particularly for guidelines.
Integration and embeddedness (mainstream)	10	21	Focus on mainstreaming of guidance into routine care but lacking detail on how to embed services across levels of the health system (particularly primary care/outpatient/community).
Local health system support	9	14	Emphasis on integration into existing health systems and services, particularly for guidelines. More limited reference to the how, including steps such as situational analysis needs assessment and planning/budgeting for the necessary infrastructure, resources, and capacity development.
Monitoring and evaluation	10	13	A need for monitoring and evaluation frameworks often mentioned but lacking in detail.
Wider multisectoral support	3	9	Minimally mentioned, with only one guideline emphasising the need for linkages to other services and systems (social assistance/protection, food, water, and sanitation) and two making brief mentions of policies/services outside of the health system. Greater presence in manuals and guidance documents but still low overall presence, along with a lack of detail when included.
Organisational capacities	3	6	Largely missing, except for a few examples that included best practice statements for, or the need to align with, emergency preparedness plans or humanitarian response strategies.

* M.M. and H.D. combined appraisal notes.

## Data Availability

The policy documents included in this review are available in the Tableau Public Platform hosted by the Emergency Nutrition Network at https://public.tableau.com/app/profile/mami.global.network/viz/MAMIScopingReview2024/Dashboard1, accessed on 19 August 2025. The database containing the policy documents (in Microsoft Excel) is available upon request from the corresponding author.

## Supplementary Materials

The following supporting information can be downloaded at: https://www.mdpi.com/article/10.3390/children12101328/s1; Table S1: Preferred Reporting Items for Systematic reviews and Meta-Analyses extension for Scoping Reviews (PRISMA-ScR) completed checklist; Supplementary Materials S2: Reflexivity statement; Figure S3a: MAMI Care Pathway: components integrated within the health system; Figure S3b: MAMI Care Pathway: mapping ‘who, what, and where’ within health services; Table S4a: Speciality of individuals contacted to source documents; Table S4b: Agency affiliation of individuals contacted to source documents; Supplementary Materials S5: Search strategy; Supplementary Materials S6: Data items; Table S7: Vulnerability factors (n = 28) mapped across 34 policy documents.

## Abbreviations

The following abbreviations are used in this manuscript:

AGREE	Appraisal of Guidelines for Research and Evaluation
BMJ	British Medical Journal
CBM	Christian Blind Mission
CCC	Clinical Care in Crises
ENN	Emergency Nutrition Network
ETAT	emergency triage, assessment and treatment
GRADE	Grading of Recommendations Assessment, Development and Evaluation
IMCI	Integrated Management of Childhood Illness
LBW	low birth weight
LMICs	low- and middle-income countries
MAMI	Management of small and/or nutritionally at-risk infants under 6 months and their mothers
MUAC	mid-upper arm circumference
NGO	Non-governmental organisation
PAHO	Pan American Health Organization
PCC	population, concept, and context
PRISMA-ScR	Preferred Reporting Items for Systematic Reviews and Meta-Analyses extension for Scoping Reviews
UN	United Nations
u6m	under six months of age
WAZ	weight-for-age z score
WHO	World Health Organization
WHZ	Weight-for-height z score
