# Supplementary material for: Invisible Pursuit: A Scoping Review of Global Policy for Continuity of Care of Vulnerable Infants Under 6 Months and Their Mothers in Low- and Middle-Income Countries
Source: Children (Basel). 2025 Oct 2;12(10):1328. doi: 10.3390/children12101328 (PMC12563175; doi:10.3390/children12101328)
Supplement: Supplementary file 1 [file children-12-01328-s001.zip › children-3857931-supplementary.pdf]

children

MDPI

Table S1: Preferred Reporting Items for Systematic reviews and Meta-Analyses extension for Scoping Reviews (PRISMA-ScR) Completed Checklist

SECTION	ITEM	PRISMA-ScR CHECKLIST ITEM	REPORTED ON PAGE #
		TITLE	
Title	1	Identify the report as a scoping review.	Included in title, p1.
		ABSTRACT	
Structured summary	2	Provide a structured summary that includes (as applicable): background, objectives, eligibility criteria, sources of evidence, charting methods, results, and conclusions that relate to the review questions and objectives.	Included in abstract, p1-2
		INTRODUCTION	
Rationale	3	Describe the rationale for the review in the context of what is already known. Explain why the review questions/objectives lend themselves to a scoping review approach.	Included in Introduction and Methods, p2-3.
Objectives	4	Provide an explicit statement of the questions and objectives being addressed with reference to their key elements (e.g., population or participants, concepts, and context) or other relevant key elements used to conceptualize the review questions and/or objectives.	Addressed population (vulnerable infants under 6 months and their mothers); concepts (characteristics, vulnerability, continuity of care); context (global policy). p2,p4.
		METHODS	
Protocol and registration	5	Indicate whether a review protocol exists; state if and where it can be accessed (e.g., a Web address); and if available, provide registration information, including the registration number.	Included reference to published national protocol that informed review objectives, methodology and appraisal. p3.
Eligibility criteria	6	Specify characteristics of the sources of evidence used as eligibility criteria (e.g., years considered, language, and publication status), and provide a rationale.	Addressed in Eligibility criteria section, p4-5
Information sources*	7	Describe all information sources in the search (e.g., databases with dates of coverage and contact with authors to identify additional sources), as well as the date the most recent search was executed.	Addressed in Information sources section, p5.
Search	8	Present the full electronic search strategy for at least 1 database, including any limits used, such that it could be repeated.	Referred to in Search strategy section, p5 and detailed in Appendix B.
Selection of sources of evidence†	9	State the process for selecting sources of evidence (i.e., screening and eligibility) included in the scoping review.	Addressed in Selection of sources section, p5-6 and in reflexivity statement, Appendix A.2 (expert opinion).
Data charting process‡	10	Describe the methods of charting data from the included sources of evidence (e.g., calibrated forms or forms that have been tested by the team before their use, and whether data	Included in Data Charting Process section, p6-7

SECTION	ITEM	PRISMA-ScR CHECKLIST ITEM	REPORTED ON PAGE #
		charting was done independently or in duplicate) and any processes for obtaining and confirming data from investigators.	
Data items	11	List and define all variables for which data were sought and any assumptions and simplifications made.	Included in Data items section, p7-8 and variables detailed in Appendix C.
Critical appraisal of individual sources of evidence§	12	If done, provide a rationale for conducting a critical appraisal of included sources of evidence; describe the methods used and how this information was used in any data synthesis (if appropriate).	Not included.
Synthesis of results	13	Describe the methods of handling and summarizing the data that were charted.	As an iterative methodology was used to navigate the data, methods of handling and summarizing the data is included in the Selection of Sources section, the Data Charting Process section, and a short synthesis of results section, p8.
RESULTS			
Selection of sources of evidence	14	Give numbers of sources of evidence screened, assessed for eligibility, and included in the review, with reasons for exclusions at each stage, ideally using a flow diagram.	See Selection of sources, p5-6 and Figure 1, Flow chart, p6. Since this was part of the process of data extraction, it is included in the Methods section.
Characteristics of sources of evidence	15	For each source of evidence, present characteristics for which data were charted and provide the citations.	Provided in both Data Charting Process section of Methods, p6-7, and in Results section, p8-19
Critical appraisal within sources of evidence	16	If done, present data on critical appraisal of included sources of evidence (see item 12).	Not applicable
Results of individual sources of evidence	17	For each included source of evidence, present the relevant data that were charted that relate to the review questions and objectives.	Policy characteristics, vulnerability factors and continuity of care dimensions are presented in the Results section and referenced in narrative and Tables, p8-19.
Synthesis of results	18	Summarize and/or present the charting results as they relate to the review questions and objectives.	Narrative synthesis is provided in the results section for each of the sub-sections that relate to the first three objectives, p8-19. The fourth objective (implications of review findings) is addressed in the discussion, p19-24
DISCUSSION			
Summary of evidence	19	Summarize the main results (including an overview of concepts, themes, and types of evidence available), link to the review questions and objectives, and consider the relevance to key groups.	p19-24
Limitations	20	Discuss the limitations of the scoping review process.	p23

SECTION	ITEM	PRISMA-ScR CHECKLIST ITEM	REPORTED ON PAGE #
Conclusions	21	Provide a general interpretation of the results with respect to the review questions and objectives, as well as potential implications and/or next steps.	p24
FUNDING			
Funding		Describe sources of funding for the included sources of evidence, as well as sources of funding for the scoping review. Describe the role of the funders of the scoping review.	Included in funding statement under Additional Information

JBI = Joanna Briggs Institute; PRISMA-ScR = Preferred Reporting Items for Systematic reviews and Meta-Analyses extension for Scoping Reviews. [20]

Supplementary Materials S2: Reflexivity statement

The authors are nutrition and health experts with experience developing global and national policy guidance (M.M., H.D., M.K., T.S.) and conducting scoping reviews (M.M., H.D., S.V.W., M.K., T.S.).

M.M. and M.K. co-conceptualised and have overseen development of the MAMI Care Pathway Package applied in this review. M.M. and M.K. co-chair the MAMI Global Network that coordinated its development through peer and expert consultation. Both M.M. and M.K. are researchers on the MAMI RISE Research Project to test the MAMI Care Pathway approach in a RCT and process evaluation in Ethiopia.

We relied on our collective expertise to consider what was feasible and useful for practice-informed policy interpretation. We have drawn upon our lived experiences in global policy processes and witnessed effects at national level to suggest constructive ways forward.

To help interpretation of our interpretations of policies, we include details of who conducted which components of the review. In the discussion, we include details of our reasonings for our findings and recommendations. We address our subjectivity in the strengths and limitations section.

Figure S3a: MAMI Care Pathway: components integrated within the health system

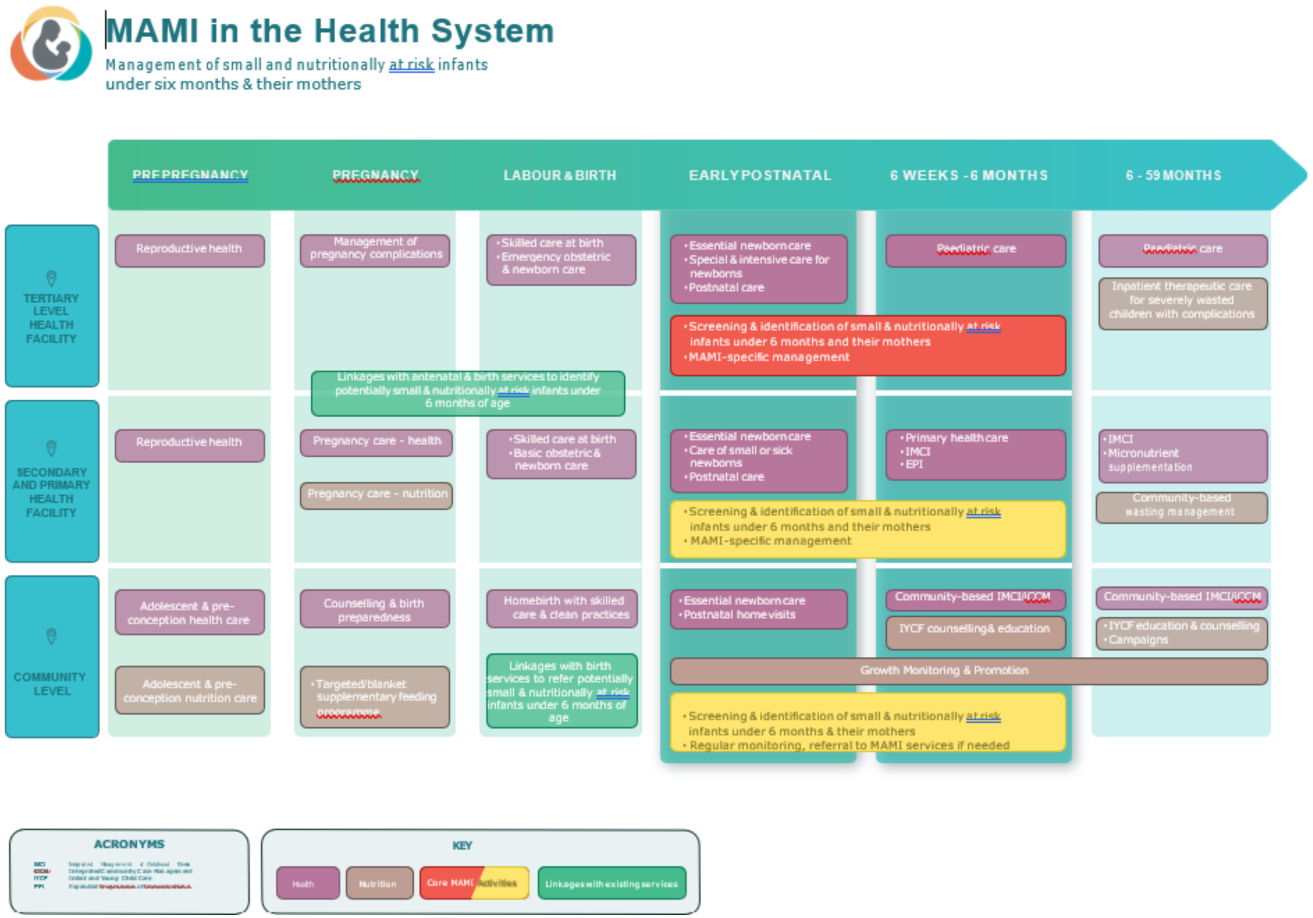

Source: MAMI Global Network, ENN, LSHTM. MAMI Care Pathway Package. v3. 2021 [24].

 https://doi.org/10.3390/children12101328

Figure S3b: MAMI Care Pathway: mapping 'who, what, where' within health services

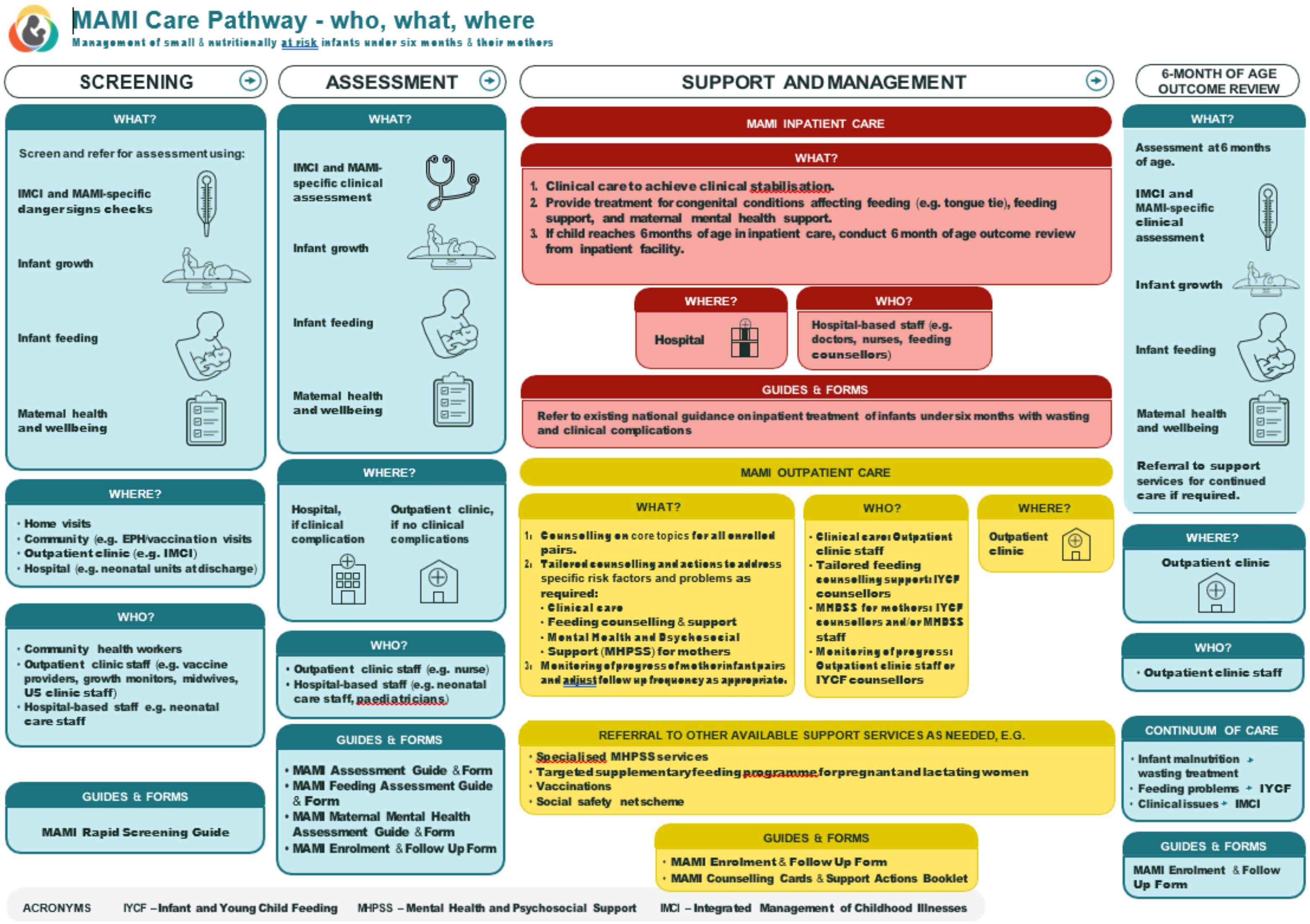

Source: MAMI Global Network, ENN, LSHTM. MAMI Care Pathway Package. v3. 2021 [24].

Table S4a: Speciality of individuals contacted to source documents

**Speciality***	**n**	**%**
Nutrition (all)	18	43%
Nutrition	16	38%
Nutrition and health	2	5%
Maternal nutrition	1	2%
Child development	4	10%
Child/Paediatric health	13	31%
Newborn	10	24%
Maternal health (all)	7	17%
Mental health	4	10%

*Note: more than one response possible and self-defined categorisation

Table S4b: Agency affiliation of individuals contacted to source documents

**Agency type**	**n**	**%**
United Nations agency	8	19%
Non-governmental organisation	19	45%
Bilateral donor (one agency)	3	7%
Foundation funder (one agency)	2	5%
Acaedemic	8	19%
Independent	1	2%
Technical organisation	1	2%
Total	42	100%

Supplementary Materials S5: Search Strategy

Search terms

newborn* or new-born or neonat* or prematur* or infant* or infancy or baby or babies or p?ediatric*

low-birth-weight or LBW or prematur* or small-for-gestational-age or SGA or small-for-age or SFA

malnourished or malnutrition or severe malnutrition or severely malnourished or severe acute malnutrition or SAM or moderate* malnutrition or moderately malnourished or moderate acute malnutrition or MAM or acute malnutrition or acutely malnourished or AM or severe wasting or severely wasted or moderate wasting or moderately wasted or wasting or wasted or thin* or stunting or stunted or growth-failure or growth-falter* or poor growth or under-weight or failure-to-thrive or FTT or failure-to-grow or growth delay or delayed growth or nutrition*deficien* or micronutrient* deficien* or nutritionally-at-risk or nutrition disorder* or protein-energy-malnutrition or PEM or development* delay or delayed development or mid-upper-arm-circumference or MUAC or weight-for-length or WFL

(1 AND 2) OR (1 AND 3) *[all vulnerable infants]*

(mother* or matern*) ADJ2 (health or nutrition or mental-health or reproductive-health or food or social-assistance or social-welfare or nutrition or malnutrition)

4 OR 5 *[all vulnerable infants OR mothers]*

policy or policies or guid* or strateg* or manual or framework or plan

6 AND 7 *[all vulnerable infants OR mothers AND guidance]*

Screen for identified documents relevant to infants under six months of age at global level

Supplementary Materials S6: Data items

Phase 1. Appraising key characteristics

The data set included the following variables:

Publication year;

URL;

Source;

Title;

Type (e.g., action plan, framework, implementation guidance, guideline, manual, report, training guide);

Aim;

Condition (disease, disorder, injury, disability, or profile of risk factors);

Target audience;

Target population (e.g., infant, mother (principal caregiver), both);

Infant's description (e.g., healthy newborn, healthy infant, vulnerable newborn, preterm infant (PT), low birth weight infant (LBW), small, sick, (non)breastfed, wasted/acutely malnourished, underweight, stunted, growth faltering, at risk of poor growth and development, feeding problem, excessive crying, disability, acute or chronic illness);

Mother's description (e.g., healthy, sick, mental health issue, malnourished, adolescent, prenatal, perinatal, postnatal issues, (not)lactating, absent, multipara, primipara, absent, dead);

Sector (e.g., child health, child nutrition, child development, maternal physical health, maternal mental health, maternal nutrition, reproductive health, newborn/neonatal health);

Level of care (e.g., tertiary, secondary, primary or community care)

Care service (e.g., maternity, child health, child nutrition units); level of care (e.g., inpatient, outpatient, community care);

Age timeline covered;

Infant risks covered (e.g., LWB, PT, SGA, growth faltering (recent weight loss, poor growth, low WAZ, low MUAC), feeding/metabolic problem, excessive crying, disability, acute or chronic illness);

Maternal risks covered (e.g., nutritional risk, impaired BF, physical health, MMH, multipara, primipara, <18 year, absent, dead);

Interventions (e.g., active case finding or screening, health assessment or IMNCI, breastfeeding (BF) assessment, non-BF feeding assessment, BF support, non-BF feeding support, clinical care, nurturing care, early childhood development (ECD), crying and sleep counselling, mental health counselling, social support, follow-up visits, home visits, family involvement, continuity of care);

Phase 2. Describing vulnerability of infants u6m and their mothers

The data set included the following variables of vulnerability:

Poor birth outcome

Small newborn:

Low birth weight (LBW) <2.5 kg; LBW <2.0 kg; very LBW <1.5 kg; extremely LBW <1.0 kg

Preterm (PT) <37 weeks gestation; very PT <33 weeks; extremely PT <28 weeks

Small for gestational age (SGA) (birth weight <10th percentile for gestational age)

Sick newborn:

Birth trauma or birth complications

Congenital illness (e.g., congenital heart disease, HIV, TB)

Disability and/or congenital abnormality (e.g., tongue-tie, cleft palate)

Macrocephaly head circumference (HC) (HC-for-age z-score >+3 or rapid crossing z lines (+1 or more z-score change in 2 months))

Microcephaly (HC-for-age z-score ≤-3)

Morbidity related to prematurity

Low infant anthropometry (including nutritional oedema)

Nutritional oedema

WAZ <-2; WAZ <-3

WLZ <-2; WLZ <-3

MUAC <115 mm for infant 6 weeks-<6months

Poor growth based on sequential measures of ponderal growth

Recent weight loss (decreasing weight; downward crossing growth lines)

No weight gain on two consecutive measurements (stationary weight)

Insufficient weight gain (flat WAZ or WLZ growth; less than 500g/kg/month)

Insufficient weight gain for preterm (less than 18g/kg/day and 0.9 cm/week in head circumference)

Risk factors for poor growth and development

Infant's health and feeding risk factors:

Infant breastfeeding (BF) difficulties (e.g., attachment, suckling reflex, refusal, intolerance)

Infant history of hospitalisation

Infant IMCI danger sign or sign of acute medical problem

Infant medical problem needing mid/long term care

Infant mental health (e.g., excessive crying)

Infant neurodevelopment concerns

Non-BF infants (e.g., unsafe preparation and use of BMS, access to BMS)

Severe ill infant and no referral possible

Mother's health, feeding, nutrition, and social risk factors:

Lack of birth spacing

Mother adolescent

Mother dead or absent

Mother's BF concerns (e.g., attachment, positioning, perceived breastmilk insufficiency mixed feeding, other ineffective feeding/time, frequency)

Mother's birth complication

Mother's MUAC

Mother's physical health (e.g., TB, HIV, disability)

Mother's social or contextual factors affecting with care and feeding (other)

Mother’s anaemia

Mother’s mental health

Multipara

Primipara

Phase 3. Appraising guidance on continuity of care

The data set included the following variables:

Condition: How is the condition or risk profile described (e.g., health problem, disease, disorder, injury, disability)?

Care across time, services and levels of care: Is care being provided across services, levels of care and time (e.g., assisting referral, providing follow-up post exit/discharge, connecting to follow-on services across health, nutrition, and social services)?

Integrated care pathway: Does care cover assessing, classifying and/or acting on the condition, including monitoring individual progress and outcomes?

Comprehensive person-centred care: Is care organised around the health needs and expectations of the person rather than on disease (including a comprehensive assessment of a person's needs and building individual resilience; involving a multidisciplinary team)?

Early childhood development (ECD): Is ECD incorporated into care?

Mother (principal caregiver), father and family support: Is the mother, father, and family engaged in care?

Community participation: Is the community sensitised (aware) and involved in the provision and organisation of care?

Embeddedness (mainstreaming in routine care): Are practices incorporated into everyday work building upon existing services (including practical re-organisation of care and staff with new roles and responsibilities; avoiding duplicative actions or vertical, disconnected service delivery)?

Local health system support: Does the policy guidance include strengthening capacities of the local health system (e.g., on governance, finances, information system, health workforce, supplies and technology, and service delivery, community participation)?

Monitoring and Evaluation (M&E): Is M&E for quality improvement comprehensively covered?

Wider multisectoral support: Are there considerations for socio-economic support and assistance (e.g., cash, food, income generation, maternity leave, childcare for working mothers)?

Organisational capacities, including resilience: Are there considerations given for developing or strengthening organisational capacities so they can better respond to (un)expected changes and absorb shocks?

Table S7: Vulnerability factors (n=28) mapped across 34 policy documents

Vulnerability factors:		Poor birth outcomes							Low anthropometry or poor growth			Risk factors related to the infant						Risk factors related to the mother											
Title of document	Condition	Congenital illness (incl. HIV, TB)	Low birth weight	Preterm	Disability or congenital abnormality	Preterm morbidity	Birth trauma or complications	Small for gestational age	Poor growth	Low anthropometry	Nutritional oedema	Breastfeeding difficulties	Illness	Not breastfed	Neurodevelopment concerns	Hospitalisation history	Mental health	Physical health	Breastfeeding conditions	Mental health	Social or contextual factors	Birth complication	Adolescent	Multipara	Absent or died	Anaemia	Primipara	Birth spacing	Low MUAC
Guideline documents (blue)																													
2023 WHO Guideline on the prevention and management of wasting and nutritional oedema in infants and children	Poor growth and development	✔	✔	✔	✔	-	-	✔	✔	✔	✔	✔	✔	✔	✔	✔	-	✔	-	✔	✔	✔	✔	-	✔	-	-	-	-

 https://doi.org/10.3390/children12101328

under 5 years																													
2022 WHO Consolidated guidelines on tuberculosis in children and adolescents	Tuberculosis	✔	-	-	-	-	-	-	✔	✔	✔	-	✔	-	-	-	-	✔	-	-	-	-	-	-	-	-	-	-	-
2022 WHO Recommendations for care of the preterm or low-birth-weight infant	Preterm, LBW		✔	✔	-	-	✔		-	-	-	✔	✔	✔	-	-	-	-	✔	-	-	-	-	-	-	-	-	-	-
2021 WHO-PAHO Evidence-based clinical practice guidelines for the follow-up of at-risk neonates	Newborn care	✔	✔	✔	✔	✔	-	-	✔			✔			✔				✔	✔	✔	-	-	-	-	-	-	-	-
2021 WHO Guideline: Infant feeding in areas of Zika	Zika virus	✔	-	-	-	-	-	-	-	-	-	✔	-	-	-	-	-	✔	✔	-	-	-	-	-	-	-	-	-	-

virus transmission																													
2021 WHO Consolidated guidelines on HIV prevention, testing, treatment, service delivery and monitoring	HIV	✔	-	-	-	-	-	-	-	-	-	-	-	-	-	-	-	✔	-	-	-	-	-	-	-	-	-	-	-
2020 WHO Improving early childhood development	ECD	-	-	-	-	-	-	-	-	-	-	-	-	-	-	-	-	-	-	✔	-	-	-	-	-	-	-	-	-
2018 WHO Guideline: Counselling of women to improve breastfeeding practices	Breastfeeding	-	-	-	-	-	-	-	-	-	-	✔	-	-	-	-	-	-	✔	✔	✔	✔	✔	✔	-	-	✔	-	-
2016 WHO Paediatric emergency triage, assessment and treatment (ETAT)	Critically ill child	✔		✔	-	-	-	-	-	-	-	-	✔	-	-	-	-	-	-	-	-	-	-	-	-	-	-	-	-
2015 WHO Recommend	Preterm	✔	✔	✔	✔	✔	✔	✔	-	-	-	-	✔	-	-	-	-	✔	-	-	-	✔	-	-	✔	-	-	-	-

ations on interventions to improve preterm birth outcomes																													
2015 WHO Guideline: Managing possible serious bacterial infection in young infants when referral is not feasible	Ill child	-	-	-	-	-	✓	-	-	-	-	✓	✓	-	-	-	-	-	-	-	-	-	-	-	-	-	-	-	-
2012 WHO Recommendations for management of common childhood conditions	Ill child	-	-	✓	✓	✓	✓	✓		✓	✓	✓	✓	✓	✓	-	-	✓	✓	-	-	-	-	-	-	-	-	-	-
2012 WHO Guidelines on basic newborn resuscitation	Newborn resuscitation	-	-	✓	-	-	✓	-	-	-	-	-	-	-	-	-	-	-	-	-	-	-	-	-	-	-	-	-	-
Guidance documents: guides (green) and																													

manuals (yellow)																													
2022 WHO Guide for integration of perinatal mental health in maternal and child health services	Perinatal mental health	-	-	-	-	-	-	-	-	-	-	-	-	-	-	-	-	✔	✔	✔	✔	✔	✔	-	-	-	-	-	-
2022 UNICEF, University of Pretoria Feeding preterm and low-birthweight newborns	Preterm, LBW	✔	✔	✔	✔	✔	-	-	-	-	-	✔	-	-	-	-	-	✔	✔	✔	✔	-	-	✔	-	-	-	-	-
2022 WHO-Europe Pocketbook of primary health care for children and adolescents	Ill child	✔	✔	✔	✔	✔	✔	-	-	✔	✔	✔	✔	✔	✔	✔	✔	✔	✔	✔	✔	✔	-	-	✔	✔	-	-	-
2022 SPOON Identifying feeding difficulties	Feeding difficulties	✔	-	-	✔	-	-	-	✔	-	-	✔	-	-	-	-	-	-	-	-	-	-	-	-	-	-	-	-	-

in infants – guidelines for healthcare professionals																													
2022 SPOON Screening children for feeding difficulties	Feeding difficulties	✔	-	-	✔	-	-	-	✔	-	-	✔		✔	✔	-	-	-	-	-	-	-	-	-	-	-	-	-	-
2022 UNICEF Integrating early detection and treatment of child wasting into routine primary health care services	Wasting	-	-	-	-	-	-	-	-	-	-	-	-	-	-	-	-	-	-	-	-	-	-	-	-	-	-	-	-
2022 WHO Operational handbook on tuberculosis. Module 5: Management of tuberculosis in children and adolescents	Tuberculosis	✔	-	-	-	-	-	-	✔	✔	✔	-	-	-	-	-	-	✔	-	-	-	-	-	-	-	-	-	-	-

2021 ENN, LSHTM MAMI Care Pathway Package, Version 3 (guiding framework)	Small and nutritionally at-risk infants and their mother	✔	✔	✔	✔	-	-	✔	✔	✔	✔	✔	✔	✔	✔	-	✔	✔	✔	✔	✔	-	✔	✔	✔	-	✔	-	✔
2020 WHO, UNICEF The Baby Friendly Hospital Initiative for small, sick and preterm newborns	Breastfeeding	-	✔	✔	✔	✔	-	✔	✔	-	-	✔	✔	✔	✔	-	-	-	✔	-	-	-	-	-	-	-	-	-	-
2020 Partners in Health, UNICEF Early Childhood Development Support for High-Risk Infants	Preterm, LBW	✔	✔	✔	✔	✔	✔	-	✔	✔	-	✔	✔	✔	✔	✔	✔	✔	-	✔	✔	-	-	✔	✔	-	-	-	✔
2019 WHO Integrated Management of Childhood Illness of the sick young infant age	Ill child	✔	✔	-	-	-	-	-	-	✔	✔	✔	✔	✔	-	-	-	-	✔	-	-	-	-	-	-	-	-	-	-

up to 2 months. Chart booklet																													
2018 IASC Field Manual on Reproductive Health in Humanitarian Settings	Reproductive health	✓	✓	✓	-	✓	✓	-	-	-	-	✓	-	✓	✓	-	-	✓	✓	✓	✓	✓	✓	-	✓	-	-	-	-
2016 WHO Oxygen therapy for children	Ill child	✓	✓	✓	-	-	-	-	-	-	-	-	✓	-	-	-	-	-	-	-	-	-	-	-	-	-	-	-	-
2015 WHO, UNICEF, USAID Caring for newborns and children in the community: planning handbook for programme managers and planners	Newborn care	✓	✓	-	-	-	-	-	-	-	-	-	-	-	-	-	-	✓	-	-	-	-	-	-	-	-	-	-	-
2014 WHO-Europe Hospital care for mothers and newborn	Newborn care	✓	✓	✓	-	✓	✓	✓	✓	-	-	✓	✓	✓	-	✓	-	✓	✓	✓	✓	✓	✓	-	-	-	-	-	-

babies: quality assessment and improvement tool																													
2014 WHO Integrated management of childhood illness – Chart booklet	Ill child	✓	✓	✓	✓	✓	✓	-	✓	-	✓	✓	✓	✓	✓	-	-	✓	✓	✓	✓	-	-	-	-	✓	-	✓	-
2014 Christian Blind Mission (CBM) Recognising impairments at birth	Newborn care	-	-	-	✓	-	-	-	-	-	-	-	-	-	✓	-	-	✓	-	-	-	-	-	-	-	✓	-	-	-
2013 WHO Pocketbook of hospital care for children	Ill child	✓	✓	✓	✓	✓	-	-	✓	✓	✓	✓	✓	✓	✓	✓		✓	✓	✓	✓	-	-	✓	-	-	-	-	-
2012 CBM Cerebral Palsy	Cerebral palsy	-	-	-	✓	✓	✓	-	-	-	-	-	-	-	✓	-	-	✓	-	✓	-	-	-	-	-	-	-	✓	-
2003 WHO Kangaroo mother care: a practical guide	Newborn care	-	✓	✓	-	✓	-	✓	✓	-	-	✓	✓	✓	-	-	-	✓	✓		✓	✓	✓	✓	✓	-	-	-	-

2003 WHO Managing newborn problems	Newborn care	✓	✓	✓	✓	✓	✓	-	✓	-	-	✓	✓	✓	✓	✓	-	✓	✓	✓	✓	✓	✓	-	-	-	✓	-	-

✓= vulnerability factor covered; - = vulnerability factor not covered; ECD= early childhood development; LBW= low birth weight; n/a= not applicable; PT=preterm.
